# Uranyl Binding to Proteins and Structural-Functional Impacts

**DOI:** 10.3390/biom10030457

**Published:** 2020-03-16

**Authors:** Ying-Wu Lin

**Affiliations:** 1 School of Chemistry and Chemical Engineering, University of South China, Hengyang 421001, China; ywlin@usc.edu.cn; Tel.: +86-734-8578079; 2 Laboratory of Protein Structure and Function, University of South China, Hengyang 421001, China; 3 Hunan Key Laboratory for the Design and Application of Actinide Complexes, University of South China, Hengyang 421001, China

**Keywords:** uranyl, metal-binding site, metalloproteins, structure-function, toxicity

## Abstract

The widespread use of uranium for civilian purposes causes a worldwide concern of its threat to human health due to the long-lived radioactivity of uranium and the high toxicity of uranyl ion (UO_2_^2+^). Although uranyl–protein/DNA interactions have been known for decades, fewer advances are made in understanding their structural-functional impacts. Instead of focusing only on the structural information, this article aims to review the recent advances in understanding the binding of uranyl to proteins in either potential, native, or artificial metal-binding sites, and the structural-functional impacts of uranyl–protein interactions, such as inducing conformational changes and disrupting protein-protein/DNA/ligand interactions. Photo-induced protein/DNA cleavages, as well as other impacts, are also highlighted. These advances shed light on the structure-function relationship of proteins, especially for metalloproteins, as impacted by uranyl–protein interactions. It is desired to seek approaches for biological remediation of uranyl ions, and ultimately make a full use of the double-edged sword of uranium.

## 1. Introduction

The growth of civilization requires the development of energy sources, including nuclear energy, which causes a worldwide concern of environmental pollution and public health. As widely used in nuclear reactors, uranium, is harmful to living systems due to both the long-lived radioactivity and high toxicity [1,2]. It is especially serious for the toxic effects on human kidneys and lung epithelial cells, such as cell necrosis [3], and osteocytic cells [4], as well as for different organisms, including plants, aquatic invertebrates/vertebrates, bacteria and fungi [5]. The most stable form of uranium under physiological conditions is uranyl ion (UO_2_^2+^), and the high toxicity of uranium might result from the ability of the UO_2_^2+^ to interact with biomolecules such as nucleotides [6] and proteins [7,8,9], resulting in an alteration or disruption of their native functions.

As reviewed previously [7,8,9], the protein crystal structures containing uranyl ions in Protein Data Bank (PDB) showed that UO_2_^2+^ is commonly coordinated by four to five O atoms, which are provided by carboxylate groups of aspartate (Asp) and glutamate (Glu), and water molecules or other ligands, such as acetate and nitrate anions. Uranyl binding to the protein may be further stabilized by hydrogen (H)-bonding interactions with uranyl oxo groups in the secondary sphere, involving the side chains or backbone atoms. It should be noted that these interactions can be exploited to design a novel artificial uranyl-binding protein with super-high selectivity and binding affinity, thereby allowing selective extraction of UO_2_^2+^ from seawater [10].

Proteins, especially for metalloproteins, play vital roles in supporting life, including electron-transfer, O_2_-binding and delivery, and catalysis, etc [11,12,13,14,15,16,17,18]. To date, a plenitude of proteins have been shown to interact with UO_2_^2+^, such as proteins in blood (human serum albumin, HSA; transferrin, Tf; hemoglobin, Hb), proteins involved in bone growth (osteopontin, OPN; fetuin-A), and the intracellar proteins (metallothionein, MT; cytochromes *b*_5_/*c*; Cyt *b*_5_/*c*; calmodulin, CaM), etc [19]. Although the structural features of some uranyl–protein complexes are well-documented [7,8,9], fewer advances are made in understanding the functional impacts of uranyl–protein interactions, with much less for other actinides-proteins interactions, as reviewed by Creff et al. very recently [20].

**Scheme 1:**
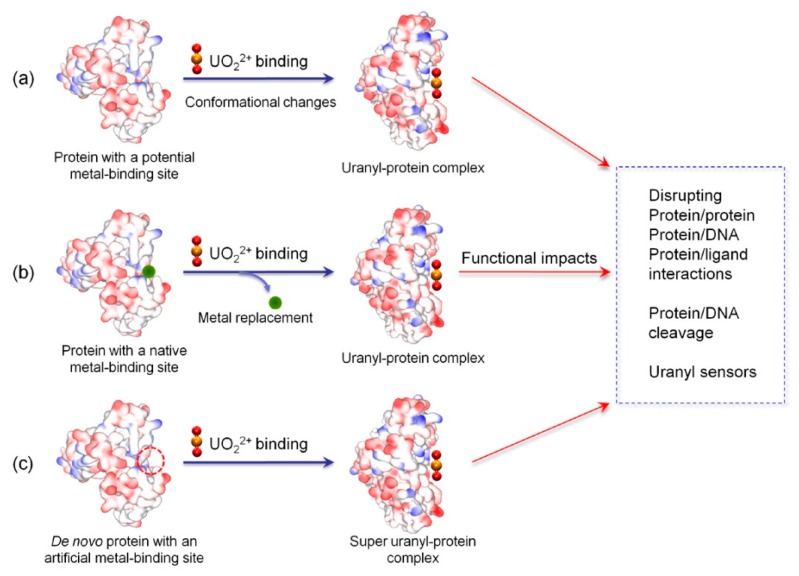
A schematic view of uranyl binding to proteins and structural-functional impacts.

Instead of focusing only on the structural information, this review highlights the recent advances in understanding the binding of uranyl to proteins, as illustrated in Scheme 1, in either potential, native or artificial metal-binding sites, or the corresponding structural-functional impacts, such as inducing conformational changes and disrupting protein-protein/DNA/ligand interactions. Future directions for studying uranyl–protein interactions are also prospected.

## 2. Uranyl Binding to Proteins

### 2.1. Binding to Potential Metal-Binding Sites

**Figure 1:**
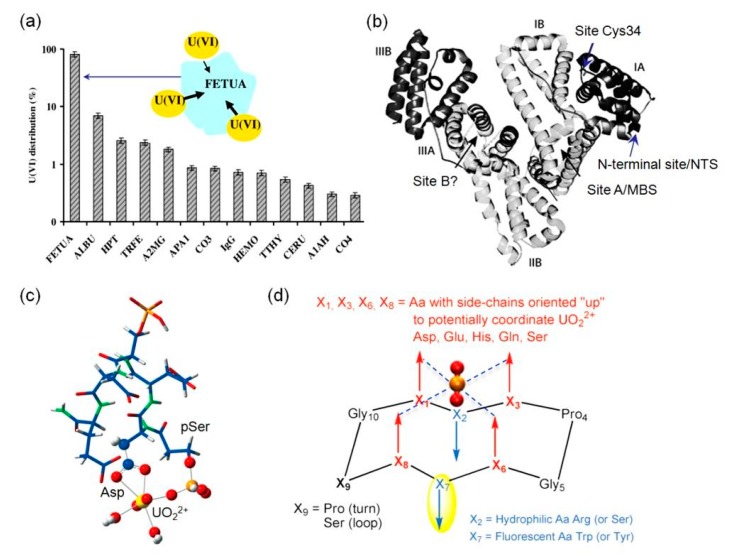
(**a**) Biodistribution of uranyl ions in serum proteins. A model of uranyl binding to fetuin-A is shown as an inset. Reprinted with permission from Ref. [22], Copyright 2013 American Chemical Society. (**b**) An overall structure of HSA showing the four potential metal-binding sites. I-IIIA and I-IIIB indicate the protein sub-domains. (**c**) A theoretical model of uranyl-peptide complex based on DFT calculation. Reprinted with permission from Ref. [28], Copyright 2013, Wiley-VCH. (**d**) Strategies for design of cyclic peptides with high binding affinity of uranyl ion. Reprinted with permission from Ref. [31] with slight modification, Copyright 2014, Wiley-VCH.

Although more than one half of all proteins are not metalloproteins and do not require metal ions for supporting their functions, they may possess potential metal-binding sites within the protein scaffold or on the protein surface [11]. According to an investigation by Vidaud et al. [21], more than ten human serum proteins are the targets of uranyl binding in blood, and not all of them are metalloproteins, such as HSA and IgG. A following study using a surface plasmon resonance (SPR) technique revealed that fetuin-A may bind more than 80% uranyl ions (Figure 1a), albeit with a concentration ~40-times lower than that of HSA in serum [22]. This is attributed to the special uranyl-binding feature of fetuin-A that it has three binding sites for UO_2_^2+^, with an apparent affinity constant (*K*_D_) ranging from ~30 nM to 10 µM, based on chromatographic and spectroscopic analysis [22]. The competitive studies of uranyl binding among fetuin-A, Tf and HSA, using isoelectric focusing coupled with inductively coupled plasma mass spectrometry (CE-ICP-MS) technique, further confirmed that fetuin-A is the preferred target in serum, with the occurrence of both fetuin-A(UO_2_^2+^) and fetuin-A(UO_2_^2+^)(CO_3_^2−^) complexes [23].

Serum albumin is the most abundant protein in blood plasma, with a concentration of 30–50 g/L. Although HSA is not a metalloprotein, it has four potential metal-binding sites (the N-terminal site, NTS; site at Cys34; site A, multi-metal binding site, MBS; and non-localized site B, Figure 1b) for various transition metals [24], and has been found to be the target of uranyl for decades [25]. Meanwhile, HSA has a lower binding affinity for uranyl (*K*_D_ = 1.7 µM) compared to that of fetuin-A (*K*_D_ = ~30 nM), and binds only ~7% of UO_2_^2+^ ions, with >80% ions bound to fetuin-A, and the remaining ions bound to Tf (see next section) and IgG, etc (Figure 1a) [22]. Szyrwiel et al. showed that addition of UO_2_^2+^ ions in foetal bovine serum formed a uranyl–protein interaction network involving 74 proteins, instead of a particular protein as the target [26].

Note that since fetuin-A also plays a role in the mineralization of bone by formation of adducts with apatite (Ca_10_(PO_4_)_6_(OH)_2_), the interactions of UO_2_^2+^-fetuin-A might be responsible for the accumulation of UO_2_^2+^ in bone tissue [27]. In addition to fetuin-A, a highly phosphorylated protein of OPN that contains a polyaspartic acid sequence, has been identified and confirmed as a target for uranyl binding in bone cells [28,29,30]. It showed that phosphorylated but not dephosphorylated OPN forms stable complexes with upon to nine UO_2_^2+^ ions for human OPN, with a *K*_D_ value of as low as 3.6 nM [29]. By studying the interactions between UO_2_^2+^ and a phosphorylated peptide (His-pSer-Asp-Glu-pSer-Asp-Glu-Val) in OPN sequence, Den Auwer and co-workers proposed one possible uranyl binding model based on DFT calculation, where UO_2_^2+^ is penta-coordinated by a bidentate carboxylate group of Asp, a phosphate group of pSer, and two water molecules (Figure 1c) [28]. These results show that both carboxylate and phosphate groups are crucial for uranyl binding and provide insights into the mechanisms of uranyl accumulation in bones.

To provide more insights into the role of phosphate groups in uranyl binding to phosphorylated proteins, Delangle and co-workers rationally designed a series of cyclic model peptides that orient four amino acid side chains containing carboxylate or phosphate groups in the same direction, such as with the “upper” face of the peptide scaffold, for potential coordination to UO_2_^2+^ ion (Figure 1d) [31]. Both acidic residues (Asp/Glu) and phosphoserines (pSer) were systematically introduced in the key positions (X1, X3, X6, and X8) of the peptide [32,33,34]. It showed that the stability of UO_2_^2+^-peptide complex increased successively by replacing the 4 Glu residues (logK = 8.2) in these positions with 1-4 pSer residues, i.e., 3 Glu/1 pSer, logK = 9.2; 2 Glu/2 pSer, logK = 10.1; 1 Glu/3 pSer, logK = 10.7; and 4 pSer, logK = 11.3. Moreover, both 1 Glu/3 pSer and 4 pSer peptides can trap two UO_2_^2+^ ions and form bimetallic complexes, suggesting a decisive role of the phosphate groups [34]. In an application research, Hu, Wang and co-workers designed a fluorescent sensor for detection of uranyl ions based on the cyclic peptide [35]. In their design, X1, X6 were coordinating residue Glu, X2, X7 were fluorescent residue Trp, and X3, X8 were pSer, respectively, which confers the sensor both high selectivity and sensitivity for UO_2_^2+^ (detection limit, 0.36 µM), and can be applied for testing river water samples with satisfactory results. For more information on the design and properties of these cyclic model peptides, the readers are referred to an excellent recent review by Garai and Delangle [36].

**Figure 2:**
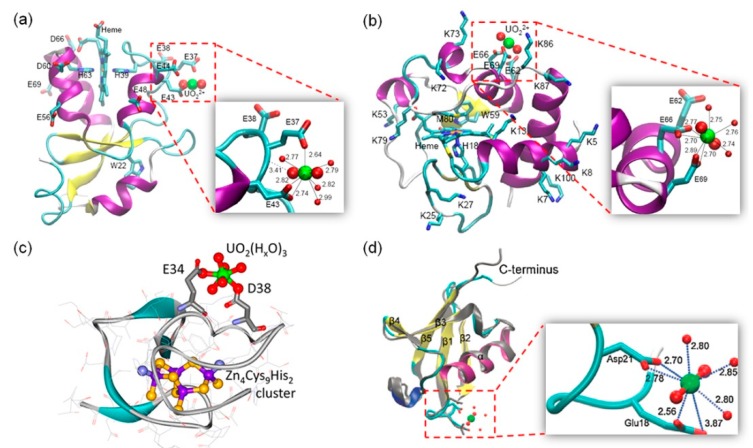
Modeling structures of UO_2_^2+^ binding to protein surface. (**a**) A model of UO_2_^2+^ binding to Cyt *b*_5_ at surface residues, Glu37 and Glu43 [44]; (**b**) A model of UO_2_^2+^ binding to Cyt *c* at surface residues, Glu66 and Glu69 [45]; (**c**) A model of UO_2_^2+^ binding to Zn_4_SmtA species. Reprinted with permission from Ref. [49], Copyright 2016 American Chemical Society; (**d**) A model of UO_2_^2+^ binding to Ub at surface residues, Glu18 and Asp21 (cyan). The structure of free Ub (gray) was shown for comparison [53]. Close-up views of the uranyl binding sites were shown as insets, highlighting the coordination and H-bonding interactions.

Heme proteins are a large class of metalloproteins that contain one or multiple heme groups and exhibit diverse functions [13,14,15,16,17,18,37,38,39]. In addition to the heme prosthetic group, other metal ions may also bind to the protein if there are potential metal-binding sites. For example, in vitro studies showed that a series of actinide and lanthanide ions such as La^3+^, Ce^3+/4+^, Th^4+^ and UO_2_^2+^ ions may bind to Hb [40]. While Ce^4+^ interacts only with the heme moiety, other ions mainly interact with the peptide ligands, causing a decrease in α-helix content. Moreover, both Th^4+^ and UO_2_^2+^ ions reduce the oxy-form of Hb at a high concentration of >100 µM [40]. Cyt *b*_5_ is a small membrane binding heme protein, and its heme-binding domain is highly negatively charged by a series of acidic residues, forming an “acidic” cluster [41,42,43]. Spectroscopic study showed that uranyl may bind to the surface of Cyt *b*_5_ with a binding affinity of *K*_D_ = 10 µM, and molecular modeling study suggested a possible uranyl binding site at surface residues, Glu37 and Glu43 (Figure 2a) [44,45]. As a biological redox partner of Cyt *b*_5_, Cyt *c* is a positively charged heme protein [46]. Meanwhile, uranyl also may bind to Cyt *c* surface at Glu66 and Glu69 (Figure 2b), as proposed by molecular modeling, albeit with a low binding affinity (*K*_D_ = 87 µM) [45]. It further showed that uranyl binding to the protein surface decreased the inherent peroxidase activity of Cyt *c*, as well as for the complex of Cyt *b*_5_-Cyt *c*, suggesting an interference of the protein-protein interactions [45].

In addition to a single metal ion or metal cofactor, metalloproteins may harbor multiple metal ions forming metal clusters that play either structural roles, electron-transfer, or catalysis functions [47]. Metallothioneins (MTs) are metalloproteins with metal-clusters and play important roles in both detoxification of heavy metals and scavenge of reactive oxygen species (ROS) [48]. Similarly, UO_2_^2+^ may bind to these metalloproteins, depending on the properties of their surfaces, and cause structural and functional consequences. Acharya and Blindauer reported that UO_2_^2+^ can bind to the cyanobacterial metallothionein SmtA, and form a heterometallic (UO_2_^2+^)_n_Zn_4_SmtA species, without alteration of the native Zn_4_Cys_9_His_2_ cluster [49]. ^1^H-NMR and molecular modeling studies suggested that UO_2_^2+^ likely bound to two surface residues, Glu34 and Asp38, with additional coordination from water molecules (Figure 2c). Although the biological consequence remained unclear, these interactions might be exploited for developing bacterial strains for bioremediation, by sequestering both soft metal ions (Zn^2+^, Cd^2+^, Hg^2+^, etc) and hard, poisonous heavy metals (UO_2_^2+^, Th^4+^, etc).

Ubiquitin (Ub) is a small protein (76 amino acids) in different organisms, which plays crucial roles in degradation of misfolded proteins via the ubiquitin proteasome system (UPS), and maintains the protein homeostasis in living cells [50]. Although Ub is not a metalloprotein, crystallographic studies showed that various divalent metal ions potentially bind to the protein, such as Cu^2+^, Zn^2+^, Cd^2+^ and Hg^2+^ [51], as well as Mg^2+^ [52]. Using molecular modeling and dynamics simulations, Lin and co-workers showed that two surface residues of Ub, Glu18 and Asp21, may coordinate to UO_2_^2+^, as well as water molecules (Figure 2d) [53]. The UO_2_^2+^-Ub complex likely has a different conjugation behavior from that of Ub, thereby affecting the UPS pathway. This prediction was also supported by experimental observations. For example, a proteomic analysis of the response of human lung cells to uranium suggested a dysfunction of the UPS system [54]. Moreover, UO_2_^2+^ is capable of crossing the blood–brain barrier [55], and Ub plays a crucial role in neurodegenerative disorders, such as in Alzheimer’s, Parkinson’s and Prion diseases [56], which provides a possibility for UO_2_^2+^ to interact with Ub in brain.

### 2.2. Binding to Native Metal-Binding Sites

**Figure 3:**
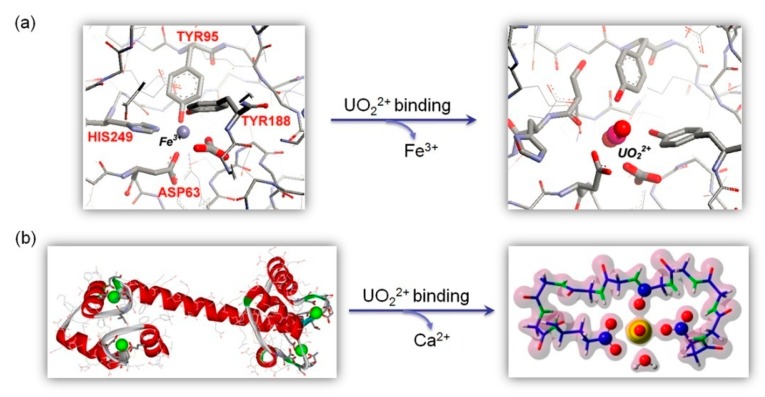
(**a**) An X-ray crystal structure (PDB code 1A8E) showing the coordination environment of Fe^3+^ in the N-lobe of Tf (left), and a proposed model of uranyl binding to the Fe^3+^ site (right) Reprinted with permission from Ref. [61], Copyright 2007 American Chemical Society. (**b**) An X-ray crystal structure (PDB code 1EXR) showing the binding of four Ca^2+^ ions in CaM (left), and a theoretical model of uranyl binding with electronic density based on DFT calculation (right). Reprinted with permission from Ref. [65], Copyright 2016 American Chemical Society.

Metal ions play crucial ions in supporting the structure and function of nearly one half of all proteins in nature, whereas there are only ten essential metal ions and most of them are transition metals, locating in the third period of periodic table [12]. The selectivity of metal ions for metalloprotein and metalloenzymes is mostly determined by the properties of ligands and their geometry [57]. Depending on the metal binding affinity in native metal-binding sites, the metal ions may be displaced by other non-native metal ions including harmful metals (Cd^2+^, Hg^2+^, Pb^2+^, etc), causing issues of human health [58]. For example, human serum Tf is an iron-binding protein responsible for iron regulation in human cells, which forms two homologous lobes at both terminals (N-lobe and C-lobe), with each lobe containing an iron-binding site [59]. An X-ray crystal structure of the N-lobe of human serum Tf revealed that the Fe^3+^ ion is coordinated by Tyr95, Tyr188, His249, and a monodentate Asp63, as well as a bidentate carbonate ion, forming an octahedral geometry (Figure 3a, left, PDB code 1A8E) [60]. Tf was also found to form a uranyl-Tf complex, and Vidaud et al. suggested that the UO_2_^2+^ ions may occupy the Fe^3+^ binding site with similar coordination ligands, except for His249 (Figure 3a, right), as indicated by the FTIR data [61]. The uranyl coordination was late on confirmed by *ab inito* quantum mechanical computational studies [62]. It should be noted that although Tf is capable of binding a uranyl ion to each of N-lobe and C-lobe, Tf was predicted to form only ~2.4% of the protein-bound uranyl in blood, due to its low binding affinity Tf (*K*_D_ = 2.8 µM) and relatively low concentration (2–4 g/L) [22].

Calmodulin (CaM) is a Ca^2+^-binding protein with four metal-binding sites and is responsible for the regulation of Ca^2+^ in biological systems (Figure 3b, left) [63]. Due to the similarity of coordination geometry between Ca^2+^ and UO_2_^2+^, UO_2_^2+^ tends to occupy the native Ca^2+^-binding sites of CaM, with even much higher binding affinity [64]. By using an extended X-ray absorption fine structure (EXAFS) technique in combination with DFT calculations, Brulfert et al. investigated the structural environment of uranyl binding to the Ca^2+^ site of CaM [65]. The results revealed that UO_2_^2+^ is coordinated by both a mono- and a bidentate carboxyl groups, as well as one carbonyl group from the main chain, with an additional ligand provided by a water molecule (pH 3, Figure 3b, right) or a hydroxyl group (pH 6). In addition to the effect of pH variation on the coordination sphere of uranyl ions, phosphorylation of CaM may increase the uranyl binding affinity. As shown by Berthomieu and co-workers [64], the phosphorylation of Thr at position 9 in the site 1 EF-hand motif of CaM, together with introduction of a Tyr at position 7, increased the binding affinity of uranyl by a factor of ~5 (*K*_D_ decreased from 25 to 5 nM) at pH 6, and by two orders of magnitude (*K*_D_ = 0.25 nM) at pH 7. The direct involvement of phosphothreonine in uranyl coordination by a monodentate phosphoryl group was further confirmed by FTIR difference spectra and EXAFS data [66]. These observations suggest a crucial role for the phosphoryl group in enhancing the uranyl binding affinity of phosphorylated proteins at physiological pH.

### 2.3. Binding to Artificial Metal-Binding Sites

**Figure 4:**
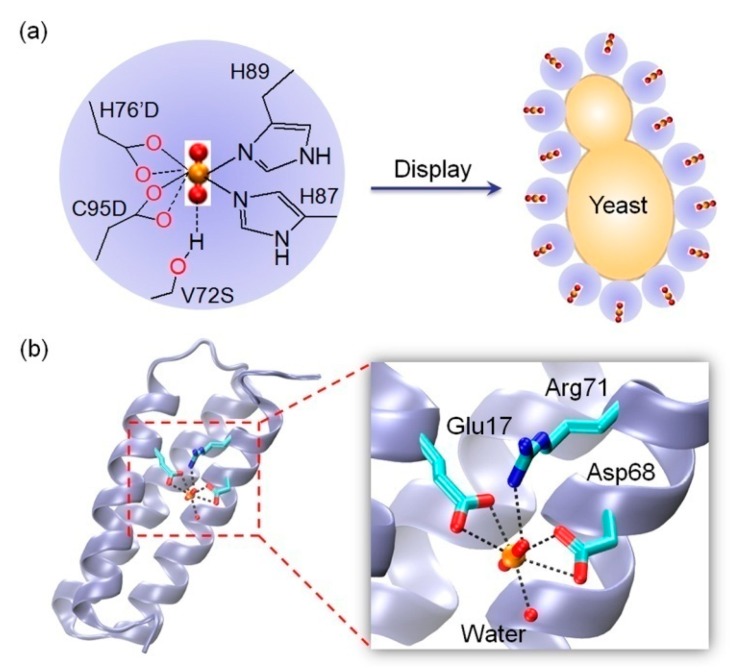
Rational design of an artificial uranyl binding site in native and *de novo* protein scaffolds. (**a**) Rational design of an artificial UO_2_^2+^-binding site in NikR by V72S/H76′D/C95D mutations (left) [67], and display of the triple mutant on yeast surface (right) [68]; (**b**) An X-ray crystal structure of a *de novo* protein with an artificial UO_2_^2+^-binding site, and a close-up view of uranyl-binding site, highlighting the coordination and H-bond interactions (PDB code 4FZP, chain B) [10].

As mentioned in the introduction, artificial uranyl-binding sites can be rationally designed in native or *de novo* protein scaffolds, based on the knowledge of the basic coordination principles of uranyl ions. In a pioneering work, He and co-workers engineered an artificial protein that binds uranyl specifically, by redesign of the Ni^2+^-binding site of a DNA-binding protein, NikR [67]. Two native ligands of Ni^2+^, His76′ from the other monomer and Cys95, were replaced by Asp, and Val72 in the secondary sphere, was replaced by a Ser that potentially provides an H-bond interaction with one oxo group of UO_2_^2+^ to further stabilize uranyl binding (*K*_D_ = 53 nM, Figure 4a, left). The results rationalized the design, which showed that the triple mutant of V72S/H76′D/C95D NikR exhibited a high uranyl selectivity, with a DNA-binding ability in presence of UO_2_^2+^ ions.

Later on, Ueda and co-workers showed that the adsorption of UO_2_^2+^ ions by V72S/H76′D/C95D NikR can be enhanced by displaying the triple mutant on the surface of yeast cells (Figure 4a, right), and the adsorbed UO_2_^2+^ ions can be recovered by treating the cells with citrate buffer at pH 4.3, which offers a convenient adsorption technique for uranyl enrichment [68]. Inspired by the uranyl coordination, Stellato and Lai recently designed three uranyl-chelating peptides that contain a DG_0-2_DG_0-2_SG_0-2_HG_0-2_H motif [69]. By modifying the peptide with a thiol group and methylene blue at N- and C-terminals, respectively, and further attaching to a gold electrode, an electrochemical sensor for uranyl ions was constructed, which exhibited a detection limit of ~50 nM, below the maximum contaminant level (MCL) for uranium of 30 µg/L (~126 nM) in drinking water [70].

In another pioneering work, He, Lai and co-workers rationally designed a *de novo* protein that binds uranyl with a super high affinity (*K*_D_ = 7.4 fM), called “Super Uranyl-binding Protein” (SUP) [10]. SUP was designed with the aid of computational screening, followed by protein engineering. An X-ray crystal structure of the uranyl-SUP complex revealed that, as computationally predicted, UO_2_^2+^ is coordinated by both Glu17 and Asp68 by bidentate carboxyl groups, with an additional coordination by water, which is further stabilized by an H-bond interaction with Arg71 (Figure 4b, PDB code 4FZP [10]). By immobilization of SUP on a solid support, such as amylose resin, the composite allows for the enrichment of UO_2_^2+^ over other metal ions. For example, it can repeatedly sequester 30–60% of UO_2_^2+^ ions in synthetic sea water [10]. In the following studies, SUP was exploited by several groups to develop new biomaterials for selective extraction of UO_2_^2+^ ions from seawater. For this purpose, SUP was either modified to form protein hydrogel microbeads, or fused with other proteins to form protein polymer or fibers [71,72,73], which increases not only the protein stability, but also the extraction capacity of UO_2_^2+^ ions, thereby making potential applications in development of nuclear energy and environmental remediation, etc.

## 3. Structural-Functional Impacts of Uranyl Binding

### 3.1. Inducing Conformational Changes

Proteins, including metalloproteins, require to adopt a proper conformation for exhibiting their functions [74]. Meanwhile, in addition to DNA molecules [75], the binding of uranyl ions to the protein or to displace the native metal ions may induce conformational changes [8]. For example, it showed that the formation of a stable complex of UO_2_^2+^-phosphorylated OPN was a result of structural changes upon uranyl binding to the protein surface [29]. The conformational change for the secondary structure was observed for bovine serum albumin (BSA) binding to uranyl, which was mainly attributed to the interaction of uranyl with carbonyl and hydroxyl groups, as determined by FT-IR spectroscopy [76]. The secondary structure was changed significantly for artificial SUP upon binding UO_2_^2+^ in solution, which is likely responsible for the discrimination of metal ions [77]. Moreover, large conformational changes occurred for the phosphorylated EF-hand motif of CaM upon uranyl binding [64].

**Figure 5:**
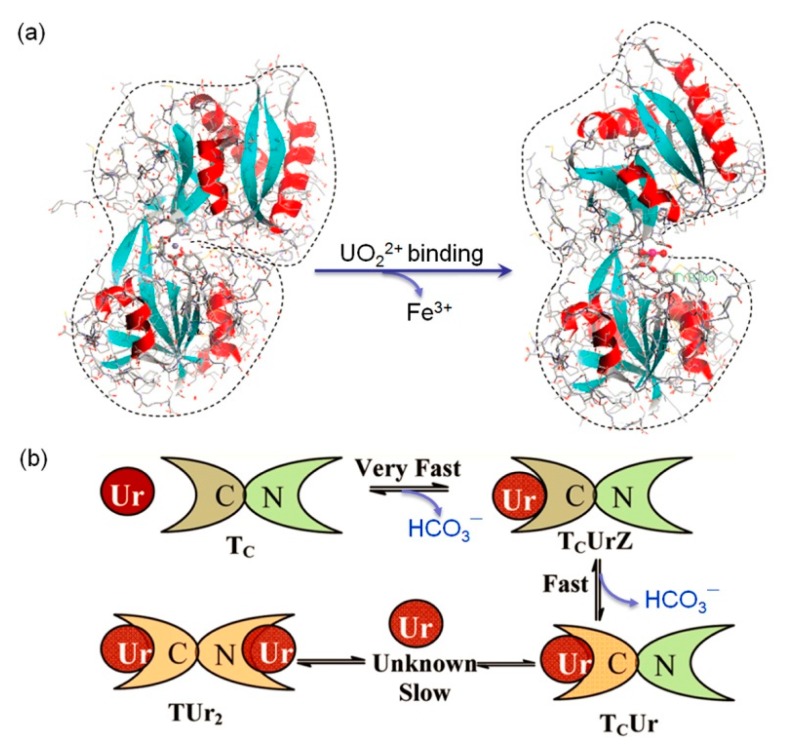
(**a**) An X-ray crystal structure of the N-lobe of holotransferrin (PDB code 1A8E) (left) and the proposed conformational changes upon uranyl binding to the Fe^3+^ site (right). Reprinted with permission from Ref. [61], Copyright 2007 American Chemical Society. (**b**) A schematic presentation of uranyl binding to Tf in several steps with different rates. Reprinted with permission from Ref. [79] with slight modification, Copyright 2011 American Chemical Society.

In case of Tf that binds two Fe^3+^ ions and forms a close conformation, Vidaud et al. showed that structural changes occurred when UO_2_^2+^ ions bound to the Fe^3+^ sites, resulting in an open conformation for the UO_2_^2+^-Tf complex (Figure 5a), whereas it is not appropriate for optimal binding to the Tf receptor [61]. Note that the major iron-acquisition system in vivo is human serum Tf and Tf receptor-1 (R_D_), which interact with each other that allows the internalization of Tf in the cytoplasm [78]. Hémadi et al. performed systematic investigations for the process of UO_2_^2+^ binding to Tf [79]. It revealed that UO_2_^2+^(CO_3_)_3_^4−^ complex first binds very fast (direct rate constant, *k*_1_ = 7.0 × 10^5^ M^−1^s^−1^) to the C-lobe Fe^3+^-binding site by loss of one HCO_3_^−^, which induces a fast (direct rate constant, *k*_2_ = 33 ± 14 s^−1^) conformation change, resulting in the loss of the second HCO_3_^−^. The resultant complex (TcUr) then undergoes two slow conformation changes (1 and 5 h), and finally forms a fully loaded uranyl complex (TUr_2_) (Figure 5b). As a consequence of conformational changes upon uranyl binding to Tf, Hémadi et al. further showed that TUr_2_ interacts with R_D_ in two steps, with each step much faster than that of the N-lobe of iron-loaded Tf (TFe_2_) interacting with R_D_, which thus allows TUr_2_ to weakly compete with the C-lobe of TFe_2_ toward interaction with R_D_ during the endocytosis process [80]. These observations provide valuable information for understanding the toxic mechanism of uranyl ions, with a possible internalization by the iron-acquisition pathway.

### 3.2. Disrupting Protein-Protein/DNA/Ligand Interactions

**Figure 6:**
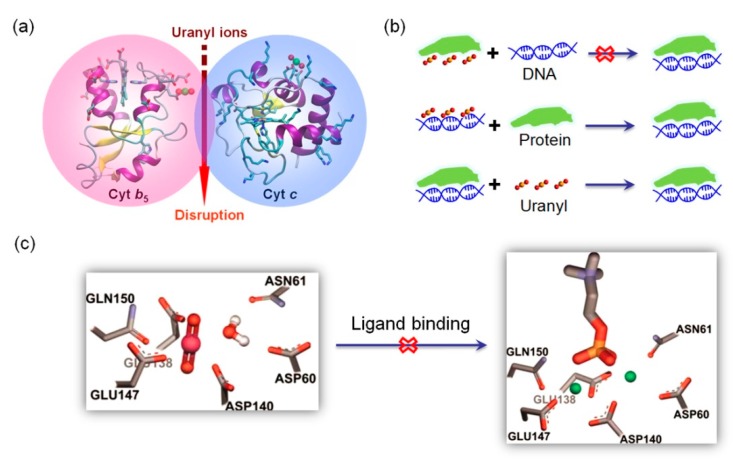
Uranyl-induced disruption of protein-protein/DNA/ligand interactions. (**a**) A schematic presentation of the disruption of Cyt *b*_5_-Cyt *c* interactions by uranyl ions, adapted from [45]. (**b**) A schematic presentation of uranyl inhibition of DNA binding to proteins and control studies [84]. (**c**) A predicted model of uranyl binding to the Ca^2+^-binding site in C-reactive protein (CRP) (left), which inhibits the binding of Ca^2+^ ions and the ligand, phosphorylcholine, as shown in an X-ray crystal structure (PDB code 1B09) (right). Reprinted with permission from Ref. [87], Copyright 2010, The Protein Society.

As mentioned in 2.1 section, Cyt *b*_5_ and Cyt *c* tend to form a dynamic protein-protein complex in biological systems [46]. Since UO_2_^2+^ tends to bind both proteins, especially for Cyt *b*_5_, the binding of UO_2_^2+^ to the protein surface may interrupt the interactions of Cyt *b*_5_-Cyt *c* complex (Figure 6a). It revealed that the uranyl binding affinity decreases from that of Cyt *b*_5_ (*K*_D_ = 10 µM) to that of the Cyt *b*_5_-Cyt *c* complex (*K*_D_ = 30 µM), which can be attributed to the dynamic electrostatic interactions between Cyt *b*_5_ and Cyt *c* such as Glu37-Lys86 that may compete the ligand of Glu37 for uranyl binding [45]. Due to the fact that the protein-protein interactions of Cyt *b*_5_-Cyt *c* complex are important in the initiation of apoptosis [46], the disruption of such interactions by UO_2_^2+^ ions may provide information for understanding uranyl-induced apoptosis [81,82].

Both protein-protein and protein-DNA interactions are important for biological systems. DNA-binding proteins such as zinc finger protein (ZFPs) play crucial roles in gene expression and DNA repair. With a common metal-binding motif in higher eukaryotes, Cys_2_-His_2_, the Zn^2+^ ion can be displaced by other metal ions such as Cd^2+^, Co^2+^, Pb^2+^, etc [83], resulting in impaired DNA binding ability. Segal and co-workers showed that UO_2_^2+^ (uranyl acetate) can inhibit the function of ZFPs by a direct interaction with the protein, instead of DNA, since no inhibition was observed for preincubation of DNA/UO_2_^2+^ and DNA/protein (Figure 6b) [84]. Moreover, uranyl acetate can inhibit other non-zinc finger DNA-binding proteins to a similar extent, suggesting a disruption of protein-DNA interaction by nonspecific protein interactions. Therefore, these observations indicate a potential mechanism for the cytotoxic and mutagenic carcinogenic effects of uranyl ions [84].

In addition to CaM, C-reactive protein (CRP) is a Ca^2+^-binding protein, and is also able to interact with other small ligands, such as phosphorylcholine (PC) and carbohydrates (D-galactose), etc, which plays multiple functions in the innate immune system in humans [85,86]. Computational analysis predicted that CRP has a high possibility to bind UO_2_^2+^ ions, and Pible et al. confirmed the prediction by biochemical experiments, with a binding affinity of UO_2_^2+^ (*K*_D_ = 0.68 µM) ~100-folder than that of Ca^2+^ (*K*_D_ = 50–78.1 µM) [87]. Moreover, as demonstrated by surface plasmon resonance (SPR) assays, UO_2_^2+^ binding to CRP was shown to prevent the Ca^2+^-mediated binding of ligand, PC (Figure 6c), which underlies some mechanisms of uranyl toxicity, albeit without in vivo results.

### 3.3. Protein/DNA Cleavage and Other Impacts

With excellent photocatalytic properties of uranyl ions [88], uranyl photocleavage has been observed not only for DNA/RNA [75,89], but also proteins, especially for those with phosphorylation, such as α-/β-casein and ovalbumin [90]. Duff and Kumar first demonstrated that UO_2_^2+^ was able to cleave a number of proteins, including BSA, HSA, porcine SA (PSA), glucose oxidase and transferrin, as induced by visible light irradiation (420–460 nm) [91]. Note that the cleavage site is highly selective, for example, the primary cleavage site on BSA is Val314-Cys315. In case of uranyl-binding proteins with phosphorylation, the photo-induced cleavage occurred in the vicinity of the phosphorylated residues, which, in turn, might be used for detecting the phosphorylation sites in proteins [90]. Moreover, the property of site-selective photocleavage of UO_2_^2+^ can be exploited to simplify the purification of recombinant proteins with a phosphorylated tag at the C-terminus, which is effectively removed upon uranyl-binding and UV-irradiation [92].

Based on DNA cleavage, Lu and co-workers [93] reported an *in vitro*-selected uranyl-specific DNAzyme for the first time, which contains a cleavage site of ribonucleotide adenosine (rA), with a critical G-A pair next to it [94], as well as a fluorosphore and a quencher at the 5′ and 3′ ends, respectively. Upon uranyl binding, the cleavage of rA resulted in release of the fluorosphore and enhancement of the fluorescence intensity, with a low detection limit (45 pM) and a million-fold selectivity of UO_2_^2+^. By attachment of the DNAzyme to the surface of gold nanoparticles (AuNP), both labeled and label-free sensors were designed for highly sensitive and selective determination of UO_2_^2+^ [95]. Moreover, the DNAzyme-AuNP probe can be modified to readily enter living cells, where it serves as an intracellular uranyl ion sensor [96]. For more sensors for the determination of uranium, the readers are referred to an excellent recent review by Wang and co-workers [97].

Additionally, due to the complexity of chemical conditions in cells, UO_2_^2+^ itself may react with other chemicals, resulting in formation of uranyl particles [98]. Lin and co-workers showed that by exposure to hepatic cells, uranyl nitrate may have transformed into precipitate of uranyl phosphate, (UO_2_)_3_(PO_4_)_2_, with contamination of other salts, which induces apoptosis by a crosstalk of the mitochondria- and death receptor-dependent pathways [82]. Similarly, the formation of uranyl phosphate particles was observed for other cells such as osteocytes upon exposure to UO_2_^2+^, which could alter the role of these cells in the bone environment [4].

## 4. Conclusions and Perspectives

In the last decades, significant progress has been made in understanding of uranyl binding to proteins in either potential, native or artificial metal-binding sites, and the corresponding the structural-functional impacts (Scheme 1). As summarized in Table 1, these proteins may have 1 to 4, and even 9 uranyl binding sites [29], with a binding affinity (*K*_D_) ranging from nM to µm. Moreover, the binding affinity can be enhanced to several fM by rational design [10]. Uranyl binding commonly induced conformational changes for the secondary structure of the proteins, whereas it may also cause large conformational changes in proteins, and interfere the protein-protein interactions [22,45,61], or even disrupt the protein-ligand/DNA interactions [84,87]. In some proteins, phosphorylation was shown to play a vital role in enhancement of the binding affinity by direct coordination of phosphate groups to the uranyl ion [29,64]. Based on this knowledge, model peptides could be rationally designed as uranyl sensors with high selectivity and binding affinity [35,36]. Owing to the unique cleavage property of uranyl ions, various uranyl-specific DNAzymes have been developed for biomedical and environmental applications [96,97].

This progress sheds light on the structure-function relationship of proteins, especially for metalloproteins, as impacted by uranyl–protein interactions. With this progress, more efforts in the future might be directed to directions as follows.

Firstly, although plenty of proteins have been discovered to date as the targets of uranyl binding [19], it is still necessary to establish new methods in combination with the advance in computational biology, for probing more potential uranyl-binding sites of native proteins in biological systems.

Secondly, although both model peptides and *de novo* proteins have been designed to bind uranyl ions specifically [10,36], it is still expected to design more artificial peptides/proteins with high stability for selective uranyl binding, and to apply them for construction of new biomaterials, or hybrid materials [99], aimed at selective extraction of UO_2_^2+^ from seawater in a large scale.

Thirdly, although some structural features of uranyl–protein complexes are well-documented [7,8,9], it is still needed to obtain more structural information for uranyl–protein complexes, not only in solid state (such as X-ray crystal structure [10]), but also in solution state (such as NMR structure [49]), for a deep understanding of uranyl-induced conformational changes.

Fourthly, although both chromatographic and spectroscopic techniques, such as CE-ICP-MS, have been developed for probing uranyl-binding interactions [22,23], it is still required to develop new techniques for identifying more protein-protein/DNA/ligand interactions impacted by uranyl binding, at the level of both metallomics and proteomics.

Finally, taking into account of the serious toxic effects of uranyl ion on living systems [3,4,5], it is desired to apply the basic knowledge of uranyl–proteins interactions for seeking approaches for biological remediation of uranyl ions, as well as other heavy metal ions.

All in all, we believe that with the development of chemical biology, especially for metalloproteomics [100], we are able to make a full use of the double-edged sword of uranium.

## Figures and Tables

**Table 1:** Uranyl binding to some proteins or peptides and the structural-functional impacts.

Protein/Peptide	Binding Site	Affinity (*K*_D_)	Structural and Functional Impacts	Refs.
Fetuin-A	3 binding sites	~30 nM–10 µM	The secondary structure was slightly modified upon binding of 3 eq. of UO_2_^2+^	[22]
HSA	4 potential binding sites	1.7 µM	Conformational changes for the secondary structure	[22,24]
Cyclic peptides	1-2 binding sites	logK = 8.2~11.3	Design of uranyl sensors	[35,36]
Phosphorylated OPN	9 binding sites	3.6 nM	Structural rearrangements	[29]
Cyt *b*_5_	1 binding site E37, E43, Wat	10 µM	Slight conformational alterations of both the heme-binding domain and the hydrophobic core.	[44]
Cyt *c*	1 binding site E66, E69, Wat	87 µM	Induces conformational changes and decreases the peroxidase activity	[45]
Cyt *b*_5_- Cyt *c* complex	Not determined	30 µM	Interference of the interactions between Cyt *b*_5_ and Cyt *c*	[45]
SmtA	1 binding site E34, D38, Water	~10^-10^ M	Very minor adjustments of either backbone or side chains	[49]
Ub	1-binding site E18, D21, Water	Not determined	Slight conformational changes and a different dynamic property	[53]
Tf	2 binding sites D63, Y95, Y188	2.8 µM	Large conformational changes and interference of the protein-receptor interactions	[22,61]
CaM	4 binding sites D1, D3, D5, E12	32 nM (pH 6)	Conformational changes for the EF-hand binding motif	[64]
Engineered CaM	4 binding sites D1, D3, D5, Y7, pThr9, E12	5 nM (pH 6) 0.25 nM (pH 7)	Conformational changes for the EF-hand binding motif	[64]
CRP	1 binding site E138, D140, E147	0.68 µM	Disrupts ligand binding to the protein	[87]
Engineered NikR	1 binding site D76, D95, H87, H89, S72 (H-bond)	53 nM	High uranyl selectivity; DNA-binding ability	[67]
*De novo* SUP	1 binding site E17, D68, Water, R71 (H-bond)	7.4 fM	Conformational changes for the secondary structure	[10,77]
